# Dynamic, adaptive and modular Digital Twin framework for resource-efficient Controlled Environment Agriculture

**DOI:** 10.3389/fpls.2026.1864757

**Published:** 2026-07-01

**Authors:** Julius Frontzek, Zühal Wagner, Stefan Streif

**Affiliations:** 1 Professorship for Automatic Control and System Dynamics, Chemnitz University of Technology, Chemnitz, Germany; 2 Department of Bioresources, Fraunhofer Institute for Molecular Biology and Applied Ecology, Giessen, Germany

**Keywords:** Digital Twin, FMI, framework, Functional Mock-up Units, hydroponics, IoT, open-source, real-time

## Abstract

In Controlled Environment Agriculture (CEA), traditional fixed set-point control is replaced by dynamic control strategies. These strategies enable joint optimization of resource use efficiency and biomass output, leverage electricity price fluctuations to reduce energy costs, and employ targeted environmental stressors to enhance crop quality and physiological resilience. Implementation of dynamic control strategies, however, builds upon real-time monitoring, robust data integration and management, and high-fidelity predictive modeling. These capabilities can be effectively provided through a Digital Twin (DT). This study introduces a novel open-source DT framework designed to support dynamic control strategies in CEA, addressing challenges in scalability, generalizability and interoperability. The framework is centered on the IoT platform ThingsBoard, providing unified, scalable data acquisition and management across heterogeneous sensor and actuator networks through vendor-agnostic integration and standardized interfaces. A significant contribution is its physics-based modeling backend, built on ordinary differential equation models developed in Modelica and exported as Functional Mock-up Units (FMUs). To ensure model accuracy across varying biological conditions, a parameter estimation pipeline is developed to calibrate and adapt these FMUs against experimental data. Building on this, a dedicated simulation backend is implemented to leverage the calibrated FMUs, providing the dynamic predictive capabilities necessary for proactive system control. Furthermore, the framework incorporates a Multirate Moving Horizon Estimation (MMHE) state estimator to estimate critical unmeasured variables, such as plant biomass. This estimator is specifically designed to handle multirate data, maintaining continuous estimates even when certain sensors provide frequent data while others are sparse or infrequent. Demonstrated through a simulation-based case study modeling lettuce growth in a vertical hydroponic farm, the DT framework’s architectural feasibility and virtual modeling capabilities are verified. Using synthetic data generated from a known true parameter set, the calibrated growth model achieved a low cross-validation prediction error, with an RMSE of 0.221g and an NRMSE of 6.62% on an independent test set. The MMHE-based state estimator effectively maintained continuous biomass estimates despite sparse synthetic measurements and model mismatch. These findings underscore the framework’s potential as a robust and extensible foundation for future physical DT implementations in CEA, enabling a 31 transition toward dynamic, data-driven, and energy-aware operations.

## 1 Introduction

The practice of vertical farming, a subfield of Controlled Environment Agriculture (CEA), represents a promising strategy for sustainable and efficient food production, especially in urban areas. It facilitates year-round cultivation through vertically stacked systems in climate-controlled environments, thereby optimizing space and resource use (Graamans et al., 2018; Kalantari et al., 2018). Nevertheless, traditional vertical farming operations often rely on static operational protocols that cannot adapt to real-time variations in environmental conditions, electricity prices, or plant responses (Kozai, 2013; Kabir et al., 2023). This restricts the overall efficiency and productivity of the farming operation (Marvin and Rutherford, 2018; Beacham et al., 2019). In order to overcome these limitations, dynamic control of climate conditions, lighting, and other process variables may be beneficial, as shown in (Kaiser et al., 2024). A promising technology for the successful realization of such control methodologies are DT, which has attracted interest for its ability to digitally replicate physical farming environments using real-time data streams. This technology can simulate, monitor, and control plant growth conditions in a predictive manner (Verdouw et al., 2021; Jones et al., 2020). However, despite the evident potential of current DT implementations in agriculture, there are notable deficiencies in scalability, generalizability, and the integration of data-driven and physics-based modeling approaches required to capture the complex interactions within vertical farming environments (Grieves and Vickers, 2017; Tao et al., 2018).

Kritzinger et al. (2018) classified Digital Twin (DT) technologies based on the direction of data flow, designating the most sophisticated class as a true DT. The defining characteristic of this class is an automated, bidirectional data flow between the physical entity and its digital counterpart. Another crucial feature of a DT is real-time connectivity (Verdouw et al., 2021; Tekinerdogan and Verdouw, 2020). Consequently, a DT framework must feature a (near) real-time data integration system tailored to the application’s specific operational requirements. While previous studies have explored digital representations for agriculture, they frequently lack the integrated, closed-loop capability required for real-time prediction and adaptive control (Jones et al., 2020). To bridge this gap, this study establishes a comprehensive framework capable of supporting both Digital Shadows (DS)—characterized by unidirectional data flow from the physical to the digital entity—and full DT functionality, which facilitates dynamic control inputs to achieve complete bidirectional operation in CEA facilities. Modern CEA facilities operate as complex networks of spatially distributed sensors and actuators across multiple subsystems—ranging from proprietary HVAC units to diverse lighting and irrigation controllers. The distributed nature of sensors is evident in the works of González et al. (2022) and Chaux et al. (2021). This technological heterogeneity creates data silos that hinder holistic optimization and the deployment of advanced control schemes, such as Model Predictive Control (MPC) or machine-learning-based optimization, which rely on continuous access to diverse sensor streams and coordinated actuation across multiple subsystems.

In response to this challenge, the present study proposes an open-source DT framework that leverages an IoT-enabled middleware layer for vendor-agnostic integration of sensors and actuators. In this regard, ThingsBoard (ThingsBoard Authors, 2026) has been selected as an open-source IoT platform solution. The framework achieves this by abstracting communication protocols and data formats into a unified, semantically consistent representation, thereby decoupling hardware from software functionality. This approach is intended to guarantee architectural scalability and enable the facility to evolve without being constrained by the technological limitations imposed by vendor-specific communication protocols. The proposed DT framework is predicated on dynamic models, which are designed to capture the complex biological and thermodynamic interactions within the CEA environment. These models are implemented in the equation-based, object-oriented modeling language Modelica, which ensures high levels of modularity and reusability across different facility configurations. In the context of facilitating an open-source workflow, the software known as OpenModelica (Fritzson et al., 2020) was employed for the development and compilation of models. In order to guarantee that the framework remains tool-agnostic and extensible, it is imperative that all services and tools are built to operate exclusively on the standardized Functional Mock-up Interface (FMI). The FMI has been extensively adopted across simulation and control platforms, thus ensuring compatibility with a broad range of existing tools (Javaid et al., 2023).

The transition from a theoretical model to a high-fidelity DT requires precise calibration, as parameters governing plant growth and heat transfer vary significantly across cultivars and architectures. We address this through a dedicated calibration pipeline that uses Maximum Likelihood Estimation (MLE) to align FMU-based simulations with experimental data, ensuring a reliable foundation for real-time decision-making. Operating the proposed DT framework under dynamic conditions requires real-time monitoring and estimation of unmeasurable variables using soft sensors to ensure safety and performance (Kabir et al., 2023). A critical bottleneck in DT implementation is the real-time acquisition of key biological states, such as plant dry biomass or transpiration rates, which cannot be measured directly without destructive or costly methods. To address this, our framework implements a state estimator as a soft-sensor service, specifically, Moving Horizon Estimation (MHE). Thus, full state information can be achieved, which is essential for advanced dynamic control schemes such as MPC and can additionally be used in anomaly detection, or Life Cycle Assessment (LCA). In order to integrate sparse and possibly delayed measurements from lab analyses, the variable-structure Multirate Moving Horizon Estimation (MMHE), introduced by Krämer and Gesthuisen (2005), was implemented. While MMHE has been shown to outperform multirate Kalman filters in estimation accuracy in bioprocess applications (Elsheikh et al., 2021), its selection here is primarily driven by its ability to natively enforce physical inequality constraints, such as ensuring non-negative biomass, which is critical for robust biological state estimation. States estimated by the DT’s MMHE service are subsequently published back to ThingsBoard. This enables their visualization through dashboards and their seamless availability to other higher-level services, such as Model Predictive Control (MPC) or anomaly detection algorithms.

DT frameworks are being used in vertical farming to improve efficiency and automation in controlled cultivation systems. However, the effectiveness of such approaches depends on their ability to address three fundamental challenges: scalability, interoperability, and human–machine interaction (HMI). Scalability is imperative to facilitate the transition from individual growth units to fully integrated vertical farms, while maintaining real-time performance and model accuracy. Concurrently, interoperability persists as a substantial impediment, as vertical farming systems depend on heterogeneous IoT devices, data formats, and control platforms that are frequently characterized by inadequate standardization (Falcão et al., 2022; Monteiro et al., 2018). Furthermore, effective HMI is required to enable operators to interact with complex system states through intuitive visualization and decision-support tools, particularly in environments where automation and data density are high (Gund et al., 2025). Recent studies in the field of controlled-environment agriculture have highlighted that most existing DT applications address these aspects only to a limited extent. These applications tend to concentrate on specific functions, such as climate or lighting control, rather than offering comprehensive, system-level solutions (Ariesen-Verschuur et al., 2022). This highlights the need for more comprehensive DT frameworks in CEA that consider scalable architectures, interoperable data infrastructures and human-centric interaction models.

The proposed framework integrates these architectural and modeling components into a unified, inter- operable DT infrastructure designed to enable scalable, continuous monitoring, estimation, and control of vertical farming environments (Monteiro et al., 2023). The incorporation of these models facilitates the (predictive) assessment of unknown system states such as crop growth, thereby enabling proactive decision-making - a combination of these capabilities is rarely observed in literature (Monteiro et al., 2018; González et al., 2022). This study fills that gap by providing a comprehensive, open-source solution that merges robust data management with modular virtual replicas. By centering the architecture on a strategic IoT core, the proposed framework represents a significant advancement toward intelligent, autonomous, and energy-aware vertical farming systems in which physiological variables inform system optimization in real-time. The remainder of this paper is organized into five sections. Section 2 provides a brief overview of related work on DTs in general as well as in CEA. Section 3 details the methodology utilized to build this DT framework, including the modeling approach, the individual components that comprise the DT framework and its overall architecture. The DT is implemented in a case study in section 4 before the framework’s design, its limitations and the case study are discussed in section 5. Finally, section 6 concludes this study.

## 2 Related work

The increasing complexity of modern entities and systems, which are subject to increasingly dynamic operating conditions, requires transformative solutions for fault tolerance detection, prediction and control. The implementation of a DT - a real-time, dynamic virtual replica of a physical system - offers a potential solution to this challenge (Classens et al., 2021). A DT is defined as a virtual model of a physical system that is created using data acquisition from sensors and operational data. The functionality of a DT encompasses the continuous acquisition of data, enabling the rapid diagnosis of anomalies (i.e. fault detection), the projection of future states or remaining useful life (i.e. fault prediction), and the incorporation of formal models and algorithms into the control loop (i.e. adaptive control). For instance, integrating physical model-based and data-driven techniques within a twin has been shown to enhance fault classification and isolation in high-precision manufacturing systems (Classens et al., 2021).

The integration of process-based models, AI-driven analytics, IoT infrastructure, and interoperable model-coupling strategies is becoming increasingly prevalent in agricultural DT architectures (Rao et al., 2026). The proposed framework aligns with this direction by operationalizing a process-based DT through FMI/FMU interoperability, middleware-based sensor integration, and real-time parameter estimation within a scalable CEA deployment architecture.

In the context of power systems, architectures incorporating digitized modeling layers have been shown to facilitate rapid identification of transformer overload and short-circuit faults, often well in advance of conventional SCADA alarms (A. Shittu et al., 2023). Beyond the scope of mere monitoring, DTs facilitate model predictive control and dynamic set-point optimization by simulating hypothetical scenarios and providing feedback to the system to modify and improve performance (Boudreau and McMillan, 2007). Consequently, as CEA systems become increasingly dynamic, characterized by variable climatic parameters, crop growth conditions, and equipment interactions, DTs offer a comprehensive platform that integrates real-time monitoring, fault detection, predictive maintenance, and intelligent control. This, in turn, enhances system reliability, energy efficiency, and crop productivity.

Multitrophic systems integrate organisms from multiple trophic levels, such as plants, microorganisms, and aquatic species, within interconnected biological and technological environments to enable efficient resource utilization and circular production processes. DT systems depend on continuous bidirectional flows of data and commands, incorporating real-time sensing, analytics, and actuation. The implementation of DTs within multitrophic and experimental research environments is inherently limited by the integration of sensors and actuators from multiple vendors, which frequently utilize incompatible communication protocols, data formats, and control interfaces. However, the utilization of heterogeneous devices poses significant challenges in terms of synchronization, semantic alignment, and unified control (Renard et al., 2024). Extensive research in the domain of smart and connected systems has repeatedly highlighted the challenges posed by heterogeneous sensor-actuator networks in facilitating real-time monitoring, coordinated control, and interoperable automation (Arshi and Mondal, 2023). These challenges are especially pronounced in academic multitrophic systems, where instrumentation from various manufacturers must be harmonized within a unified cyber-physical framework. The management of such technological heterogeneity necessitates the implementation of sophisticated integration strategies to ensure reliable data exchange and system synchronization (O’Connell et al., 2023). Furthermore, the interoperability of DT ecosystems is constrained by the absence of standardized interfaces and architectures. In many cases, developers are confronted with *ad hoc* alignment of APIs rather than true composability across systems of systems (David et al., 2024). It is therefore imperative to address these integration and interoperability barriers if the full potential of DTs in multitrophic systems is to be realized, thus enabling robust fault detection, accurate prediction, and tightly closed-loop control across multiple trophic levels.

The objective is to facilitate seamless integration of diverse tools, sensors and actuators from multiple vendors. An open framework facilitates the modular incorporation of simulation, analytics, and control tools, while providing standardized interfaces for heterogeneous devices. This enables seamless communication between sensors, actuators, and the DT core. This extensibility is of particular importance in multitrophic systems, where multiple subsystems with distinct physical, biological, and computational characteristics require synchronization and collaborative control. Conventional DT implementations are frequently restricted by non-standard interfaces, proprietary ecosystems, and heterogeneous hardware, thereby constraining interoperability, data exchange, and coordinated control (Renard et al., 2024; David et al., 2024). In order to address these challenges, there is an imperative requirement for open, as well as extensible, DT frameworks. The adoption of open and modular architectures by DT platforms has been demonstrated to engender greater flexibility, scalability, and reusability, thereby facilitating cross-disciplinary experimentation, predictive analytics, fault detection, and adaptive control in complex multi-vendor environments (O’Connell et al., 2023; Arshi and Mondal, 2023). It is evident that the establishment of such open and extensible frameworks is a necessary step toward the realization of fully integrated, resilient, and adaptive DTs for multitrophic systems. This provides a foundation for innovation and reliable system operation (Renard et al., 2024).

## 3 Methods

### 3.1 Modeling

The proposed framework utilizes a state-space modeling approach to describe the essential dynamics of CEA. While these models are often derived from first principles, the formulation presented here is generalizable to data-driven models such as ARX models that can be expressed in state-space notation. As demonstrated by Padmanabha et al. (2023), a high-fidelity CEA model must account for the bidirectional feedback between the physical infrastructure, which we refer to as the production unit, and the cultivated species. Therefore, the following two interconnected subsystems are considered: (i) the thermodynamic processes of the production unit and (ii) the biological processes of the cultivated species. The production unit governs the transport of heat and matter (typically in air and water) and is influenced by both controllable actuators and external disturbances such as outdoor climate conditions. The species dynamics may comprise crop-specific processes, including growth, development, and heat/matter exchanges with the production unit. Its processes are dependent on the environmental conditions established by the production unit and, in turn, interact with it through the exchange of heat and matter (e.g., *CO*_2_, water vapor, and latent heat). Collectively, these coupled subsystems form a closed loop wherein the production unit provides the environmental context for plant growth, while the plants continuously influence the microclimate through their metabolic activity.

To formalize the interaction between the production unit and the biological processes, the system is represented as a coupled set of first-order differential equations. Let *x_u_* ∈ *R^nu^* and *x_s_* ∈ *R^ns^* denote the state vectors for the production unit (e.g. air temperature, relative humidity) and the cultivated species (e.g. biomass, carbon content), respectively. The combined system dynamics can be expressed in the general form:

(1) $$\dot{x}(t)=f(x(t),u(t),d(t),\theta )$$

(2) y(t)=h(x(t),u(t),d(t),θ),

Where $x(t)={\left[x_{u}^{T},x_{s}^{T}\right]}^{T}$ is the augmented state vector, u(t) represents the controllable inputs (e.g. HVAC setpoints, LED intensity), d(t) represents uncontrollable disturbances (e.g. solar radiation, outdoor enthalpy), $\theta \in R_{p}$ is the parameter vector consisting of e.g. heat transfer coefficients, light-use efficiencies, etc. and y(t) is the observation vector containing measurable outputs.

In the context of model development, the high-level modeling language Modelica is utilized in this study for multi-physical systems modeling. Its object-oriented nature facilitates the construction of modular and hierarchical models, thereby enabling the reuse and extension of submodels across a range of applications. The open-source Modelica-based modeling software OpenModelica has been chosen for the implementation and simulation of models in this study, which also facilitates the export of models as FMUs for subsequent integration into the DT’s parameter estimation pipeline as well as state estimation and control services. This workflow decouples model formulation from its execution environment, ensuring that the same physical model can be readily integrated into all other tools of the framework.

With this grey-box formulation, the DT model’s performance depends heavily on the calibration of the parameter vector *θ*. While some parameters are derived from literature or physical constants, others are specific to the CEA facility or cultivated species and may shift over time. This necessitates a robust parameter estimation pipeline, as detailed in the following section, to align unknown parameters such as kinetic constants and thus the models themselves with real-world sensor data. It may also be used by operators to periodically re-estimate parameters, allowing for manual updates to the models integrated into the DT.

#### 3.1.1 Parameter estimation

The formulated dynamic models typically contain unknown parameters *θ*, such as kinetic constants, thermodynamic properties, or empirical relations. Estimation of these parameters is therefore required before these models can be used within the DT framework’s services. Although some can be obtained from technical data sheets or the literature, others must be fitted to experimental measurements to ensure that the model reproduces the measured system behavior.

To achieve this objective, a parameter estimation pipeline has been developed using Maximum Likelihood Estimation (MLE), assuming independent and identically distributed Gaussian measurement noise. To handle large datasets, a data-preprocessing stage has been implemented, in which the measurement data are first filtered with a fourth-order Bessel filter before being downsampled to a lower frequency. Parameter estimation is conducted on auto-validation datasets and later evaluated on cross-validation datasets to assess how well the fitted model predicts dynamics on unseen data.

The implementation of the parameter estimation pipeline follows the approach proposed by Cañas et al. (2023), in which the FMU is imported into the pipeline using CasADi (Andersson et al., 2018), as described in Andersson (2024). However, Cañas et al. (2023) have noted that variables declared with the Modelica variability keyword ‘parameter’ are treated as compile-time constants when exported as an FMU from OpenModelica and thus cannot be redefined as optimization variables in CasADi. To overcome this challenge, these parameters were defined as ‘input’ in the Modelica source code and later automatically redeclared as parameters in the parameter estimation pipeline. After exporting the model as an FMU and importing it through CasADi’s DaeBuilder interface, these input variables are re-declared as parameters in the Python pipeline, thereby restoring their ability to be optimized.

The MLE problem is formulated using a direct multiple-shooting scheme, where the optimization decision variables include the parameter vector *θ* and the state vectors **x***_d,k_*at each shooting node *k* for every dataset *d*. This approach ensures the discrete-time state transition function denoted as **F**(**x***_d,k_*,**u***_d,k_,θ*) maintains continuity through equality constraints and has been shown to outperform single-shooting and collocation approaches in computational efficiency (Cañas et al., 2023). The state transition function is built using CasADi integrators that utilize the solvers CVODES and IDAS. The resulting Non-Linear Programming (NLP) problem is solved using IPOPT (Wächter and Biegler, 2006).

**Figure 1:**
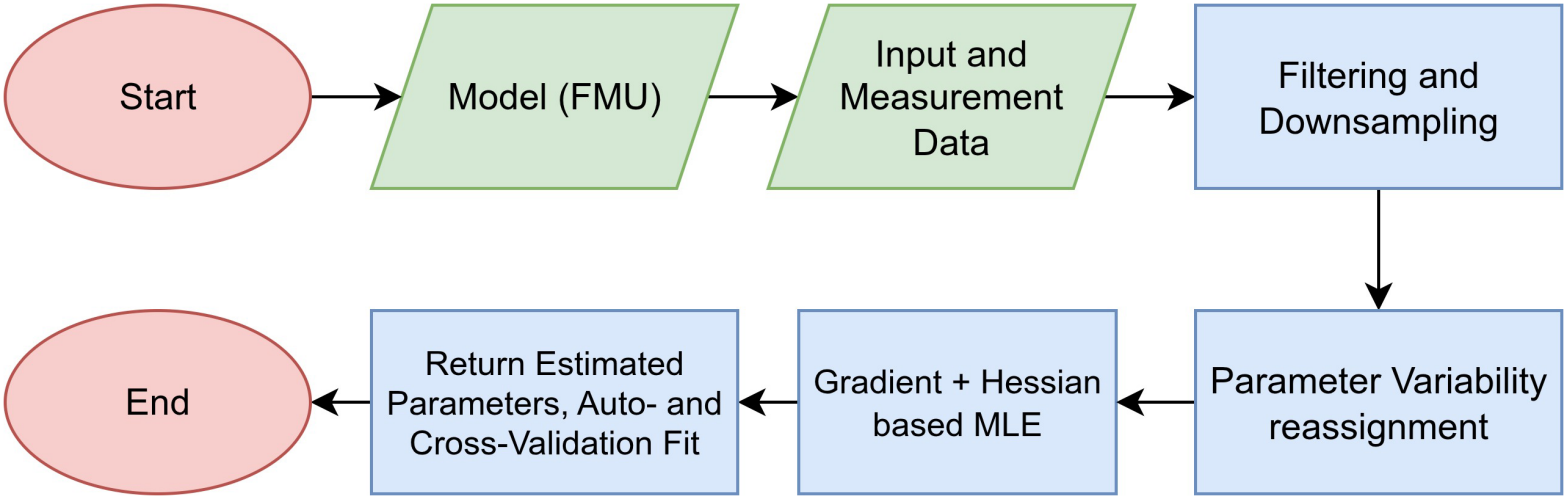
Parameter estimation pipeline: Inputs are shown in green while process steps are depicted as blue boxes.

The pipeline manages heterogeneous, sparse measurement data from multiple sensors operating at non-uniform sampling rates through the mapping matrix **M***_d,k_*, which acts as a selection operator to extract only the available observations at time step *k*. This matrix is used to align the model output function **h**( $x_{d,k}$, $\;u_{d,k}$) with the available measurements $y_{d,k}$, where $u_{d,k}$represents the known system inputs. To ensure model robustness and prevent overfitting, the architecture facilitates simultaneous calibration across multiple experimental datasets, enabling rigorous auto- and cross-validation. Assuming additive white Gaussian noise for the measurements, the MLE objective function is defined by Equation 3 in which the residual vectors *r* between measurements **y** and model outputs **h** are minimized across all auto-validation datasets *D* and their discretized time steps *N_d_*. These are weighted based on the time step and dataset-dependent measurement matrices $R_{d,k}$as well as the matrix $W_{\text{count}}$ accounting for the number of measurements to increase weight in sparse measurement channels. State vectors at the start of each dataset $x_{d,0}$ are also optimized with deviations from an initial guess ${\hat{x}}_{d,0}$ being penalized via the matrix **Q**_0_. Box constraints for state and parameter values are imposed via $x_{\text{min}}$≤ $d_{d,k}$≤ $x_{\text{max}}$ and $\theta_{\text{min}}$ ≤ *θ* ≤ $\theta_{\text{max}}$ respectively to ensure physical feasibility. The workflow of the parameter estimation pipeline is visualized in **Figure 1**.

(3) $$\begin{array}{ll} \underset{x_{d,k},\theta }{\min}\;J(\theta ,X) & =\sum_{d=1}^{D}[\sum_{k=0}^{N_{d}}r_{d,k}^{⊤}(R_{d,k}^{-1}W_{\text{count}})r_{d,k}+{\left(x_{d,0}-{\hat{x}}_{d,0}\right)}^{⊤}Q_{0}(fx_{d,0}-{\hat{x}}_{d,0})]\\ \text{s}.\text{t}.\;x_{d,k+1} & =F(x_{d,k},u_{d,k},\theta ),\;k=0,\ldots ,N_{d}-1,\\ r_{d,k} & =M_{d,k}(y_{d,k}-h(x_{d,k},u_{d,k})),\;k=0,\ldots ,N_{d},\\ x_{\min\;\;} & \leq x_{d,k}\leq x_{\max\;\;},\\ \theta_{\min\;\;} & \leq \theta \leq \theta_{\max\;\;},\\ \forall d\in \{1,\ldots ,D\} \end{array}$$

##### 3.1.1.1 Evaluation metrics

Quality of fit is further evaluated using Root Mean Squared Error (RMSE) and Normalized RMSE (NRMSE) for both the auto-validation and cross-validation datasets. Sparse measurements are accounted for by excluding measurement channels.

(4) $$J_{i}=\{j\in \{1,\ldots ,N\}|y_{ij}\text{ is not NaN}\},\;N_{i}=|J_{i}|,$$

(5) $$\text{RMSE }\;=\sqrt{\frac{1}{n_{y}}\sum_{i=1}^{n_{y}}\frac{1}{N_{i}}\sum_{j\in J_{i}}{\left(y_{ij}-h\left(\theta ,x_{ij}\right)\right)}^{2}}$$

(6) $$\text{NRMSE }\;=\frac{1}{n_{y}}\sum_{i=1}^{n_{y}}\frac{\sqrt{\frac{1}{N_{i}}\sum_{j\in J_{i}}{\left(y_{ij}-h\left(\theta ,x_{ij}\right)\right)}^{2}}}{\max_{j\in J_{i}}\;y_{ij}-\min_{j\in J_{i}}y_{ij}}.$$

These metrics provide a quantitative measure of the model’s predictive accuracy and generalization capability. The resulting parameterized models can subsequently be deployed within the DT framework for state estimation, prediction, and control applications.

### 3.2 Proposed DT framework: architecture and component integration

**Figure 2:**
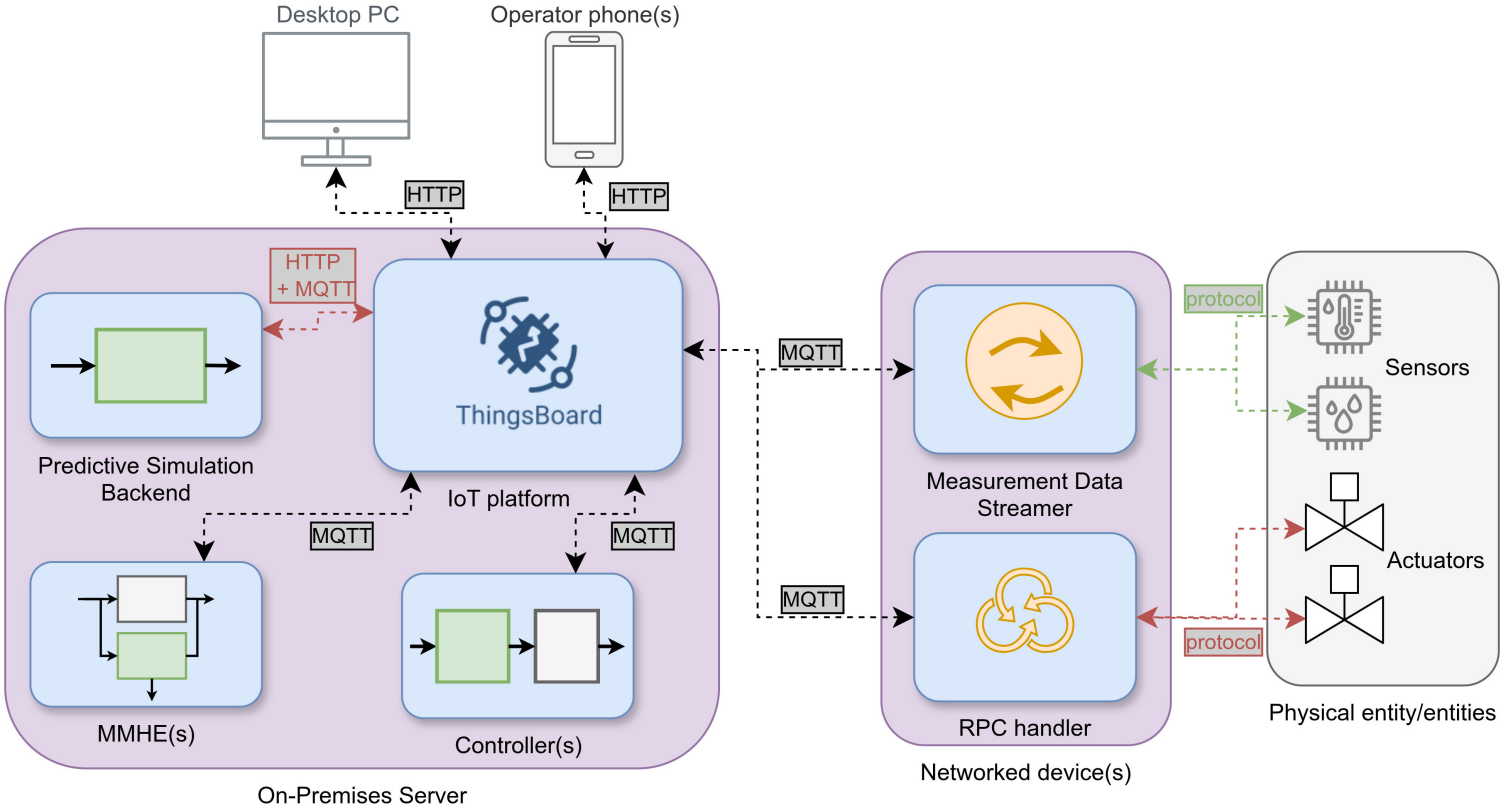
General architecture of the proposed DT framework.

The present study proposes an architecture comprised of several hardware and software components to implement a DT for existing CEA facilities. It features a central server, deployable either on-premises or in a cloud environment, that hosts the IoT platform and its database. Furthermore, it is proposed that all additional services, such as the predictive simulation backend and MMHE, be hosted on this central server. An arbitrary number of networked devices are used for bidirectional communication with the IoT platform to relay measurements and actuation commands. Human-machine interaction is enabled through the ThingsBoard Web UI and a mobile app. Communication between the IoT platform and the services both on the server and on the networked devices is realized via the MQTT protocol. The predictive simulation backend also communicates via HTTP, as described in 3.2. Crucially, no specific communication protocol is dictated between measurement data streamers and sensors to facilitate a vendor-agnostic setup. This also applies to the RPC handlers and their connected actuators. **Figure 2** presents a general overview of the framework’s architecture. The utilization of blue boxes denotes services hosted by the devices depicted in purple. The grey box indicates the physical production facility in which sensors and actuators are located. The core infrastructure is detailed below through its functional software components:

#### 3.2.1 IoT platform

The DT architecture is centered on the open-source IoT platform ThingsBoard (ThingsBoard Authors, 2026), functioning as the central integration layer for all sensors, actuators and DT services. In addition to a paid professional edition, it offers a free community edition and can be self-hosted. All data is stored in its integrated PostgreSQL database.

The selection of ThingsBoard as the core IoT platform is supported by the findings of the evaluation conducted by (Turki et al., 2024) and the review by (De Nardis et al., 2022). The selection criteria, which included horizontal scalability, extensive support for communication protocols, an open-source nature, a straightforward setup process, and dashboard and analytics functionalities, all point to ThingsBoard as the optimal choice. This decision is further supported by the findings of studies such as those by (Di Felice, 2023) and (Ismail et al., 2018).

#### 3.2.2 Measurement data streamer

Measurement data streamers (MDS) serve as lightweight, sensor-specific programs that continuously and asynchronously collect measurement data from connected sensors and transmit it to the central IoT platform via MQTT. To mitigate intermittent network failures, each streamer can be set up to maintain a local buffer for temporary storage, publishing both buffered and newly acquired measurements once connectivity is restored. Each MDS is custom-built and must adhere to the interfaces and communication protocols of its respective sensors. Thus, they enable flexible integration of heterogeneous sensor types, including cost-effective and readily available sensors commonly used in research and small-scale industrial environments.

#### 3.2.3 Remote Procedure Call handler

To relay actuation commands from the DT to physical actuators (DT2PT communication), the framework employs Remote Procedure Call (RPC) handlers that bridge the IoT platform with vendor-specific actuator protocols. These actuation instructions may be initiated manually through user dashboards or automatically via control algorithms, and are sent to ThingsBoard as RPC requests targeting particular actuators. RPC handlers are lightweight, actuator-specific programs that continuously monitor the IoT platform for incoming RPC requests. Upon receiving a command, the handler translates the standardized ThingsBoard RPC message into the vendor-specific communication protocol required by the target actuator (e.g. Modbus, BACnet, proprietary APIs) and executes the actuation.

Thus, a vendor-agnostic interface is realized on the ThingsBoard side, which abstracts the implementation details of individual actuators from higher-level services. Multiple RPC handlers can be deployed across spatially distributed network devices within the facility network, that each relay actuation commands for a subset of actuators. By encapsulating protocol-specific logic within individual handlers, the framework accommodates arbitrary communication protocols without modifying the core DT architecture, ensuring architectural scalability and flexibility as new actuator types are integrated.

#### 3.2.4 Predictive simulation backend

Integrating a predictive simulation backend into the DT is essential to transition from passive monitoring capabilities to active, predictive optimizations. Thus, the DT may be used to estimate how crop growth trajectories and other modeled properties may evolve over time.

The predictive simulation backend architecture is designed for modularity and interoperability, relying on two key components for each (sub-) system: (i) a Functional Mock-up Unit (FMU) containing the physics-based model, and (ii) a YAML configuration file that specifies the integration between the model and the IoT platform. The YAML configuration file serves multiple purposes: It defines the mapping between FMU variables (states, inputs, outputs) and their corresponding ThingsBoard telemetry keys, specifies simulation settings such as the numerical solver and time discretization, and provides model parameter values that may differ from the FMU defaults or require facility-specific calibration. This approach enables model calibration without modifying the underlying FMU files, thus decoupling modeling from deployment. Further, it is specified whether the model shall be simulated using the most recent inputs (thus assumed constant across the simulation horizon) or using predicted input trajectories stored in ThingsBoard. The YAML configuration file for the case study is available in **Appendix**.

At each prediction cycle, the backend retrieves the current state and input values from ThingsBoard via the ThingsBoard Python REST client (ThingsBoard Team, 2026b). The FMU is then simulated forward in time using CasADi and the specified solver, generating predicted trajectories for all model outputs and states. These predictions are published to dedicated virtual devices in ThingsBoard via the ThingsBoard Python Client SDK (ThingsBoard Team, 2026a), making them accessible to monitoring dashboards, control algorithms, and other DT services. A key design decision for this service was to operate asynchronously across all subsystems and thus non-blocking and parallel for scalability. This includes both data transfer to and from ThingsBoard and the simulations themselves and was implemented using Python’s *asyncio* library.

To ensure consistency and enable temporal comparison, all predictions are published to a fixed time grid aligned with the prediction horizon. Thus, when a new prediction is generated, its previous trajectory is automatically overwritten.

#### 3.2.5 Multi-rate Moving Horizon Estimation

By incorporating model-based state estimators such as MMHE, the DT framework offers a scalable solution to the inherent challenge of limited sensor availability in CEA production units, establishing a robust foundation for advanced monitoring and predictive control. This is particularly important for advanced control algorithms such as MPC, which rely on full state information.

Although its optimization-based formulation imposes substantial computational demands, especially for large models and extended MMHE horizons, MMHE remains practical when executed on a centralized, high-performance server, as envisioned in our framework. The variable-structure MMHE formulation is adopted from Elsheikh et al. (2021).

#### 3.2.6 Controllers

To increase profitability in CEA, optimizing the cultivation process is of great importance. To this end, actuators that influence environmental conditions should be controlled to minimize energy and other costs while maximizing biomass output. Various research papers have studied optimal control strategies for indoor cultivation of plants and other organisms. Since many of these approaches rely on measurement and state estimator data from various sources, data centralization in our DT framework can help to facilitate implementation of these strategies. We propose implementing controllers that stream data from the central IoT platform and send their computed control inputs back to the platform via RPC requests. These are then relayed to the respective actuators via the previously described RPC handlers.

#### 3.2.7 Human-machine interface

While the MDS and RPC handlers ensure that the DT remains synchronized with its physical counterpart, the insights generated must be actionable for the operator. To this end a Human–Machine Interface (HMI) for the DT can be implemented via interactive dashboards in ThingsBoard. The platform provides a wide range of built-in widgets that enable comprehensive monitoring of the physical twin, including status indicators, SCADA-style visualization elements and time-series charts. The latter can e.g. be used to visualize predictions by the simulation backend. Moreover, manual actuator control can be achieved through configurable control widgets that transmit desired actuation commands to the corresponding RPC handlers via remote procedure calls (RPCs).

## 4 Results

### 4.1 Case study: DT implementation for a vertical lettuce farm

To demonstrate the workflow, data integration capabilities, and computational feasibility of the proposed framework, an entirely simulation-based case study was implemented for vertical farming. All validation metrics and estimator evaluations presented herein rely on synthetic data streams to establish a controlled proof-of-concept before physical deployment. Specifically, lettuce cultivation was undertaken within a commercially available small-scale hydroponic vertical farming system designated the *GreenResearcher* (GR). This system comprises multiple actuators, including nutrient pumps, lights and ventilators as well as sensors such as pH, EC, CO2, etc. The GR was placed in a climate tent equipped with an air-conditioning unit. The installation of additional sensors was undertaken to measure further climatic conditions inside and outside the climate tent, as well as fresh biomass of the cultivated lettuce. The additional sensors included temperature, *CO*_2_, and relative humidity sensors inside and outside the climate tent and light intensity sensors next to the lettuce. It is important to note that all of these systems operate on a 15-second sampling period. Integrating this diverse array of sensors into the DT served to rigorously validate the framework’s scalability and its capacity for vendor-agnostic communication across heterogeneous data streams. However, for the specific case study presented herein, only light intensity, *CO*_2_, and air temperature were utilized as the driving inputs for the crop growth model, while the remaining parameters were reserved exclusively for baseline monitoring and future modeling extensions.

**Figure 3:**
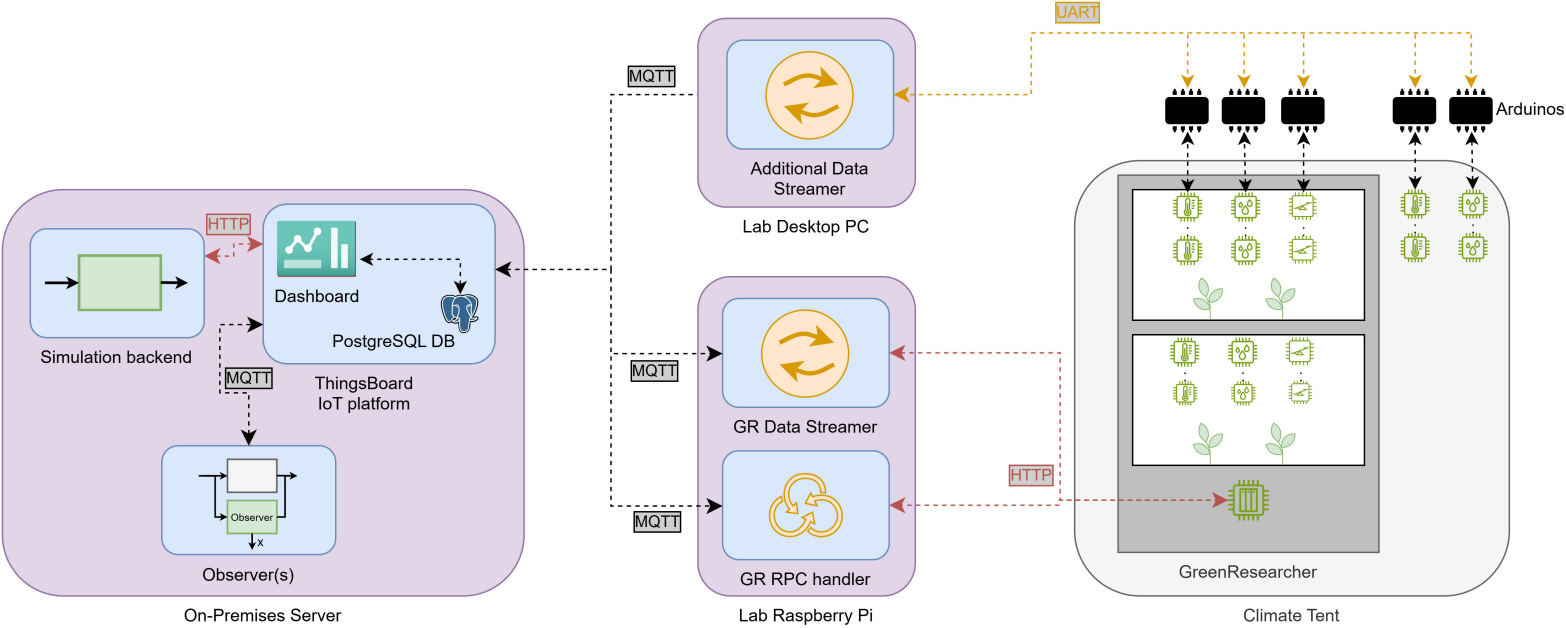
Server with services, MDS with Raspberry Pi, and climate tent.

The DT architecture for the case study was adopted from section 3.2, as illustrated in **Figure 3**. In addition to an on-premises server, two networked devices were configured to host MDS and RPC handlers. The two networked devices in particular were a desktop PC and a Raspberry Pi. The GR sensor and actuator data were retrieved using a custom MDS connected to the GR via its HTTP endpoints. Furthermore, the manipulation of the GR’s actuators was realized via an RPC handler, which also communicated with the GR via its HTTP endpoints.

#### 4.1.1 Lettuce growth model

The biomass growth dynamics of the lettuce plants were modeled in alignment with the methodology proposed by van Henten (1994). The model consists of a single state, namely the dry matter content of biomass *W*. Its dynamics are a function of light intensity *I*, carbon dioxide concentration *C*, as well as air temperature *T*, as described by (7). Plant *CO*_2_ consumption and transpiration were modeled in the original work but were neglected in this case study. This level of simplification is appropriate because the primary objective of this study is to verify this framework’s software architecture and FMU interoperability rather than to present a novel or highly detailed plant physiological model. Furthermore, while mature, field-scale crop models (e.g., DSSAT, APSIM, or AquaCrop) are widely used in agriculture, they are typically tailored for daily time steps and outdoor environmental conditions. In contrast, the chosen model is explicitly suited for tracking high-frequency indoor cultivation dynamics. Its compact structure enables it to be embedded directly into control applications with high sampling rates and seamlessly integrated into real-time state estimation frameworks, such as the MMHE pipeline demonstrated herein. While said vital physiological processes are omitted here, the framework’s modular FMI-compliant design explicitly ensures that more complex, multi-state models can be seamlessly swapped in without modifying the core architecture.

(7) $$\frac{dW}{dt}=c_{a,med}(c_{\alpha \beta }{\text{Φ}}_{CO_{2},a\_c}-c_{W,c\_a}\cdot W\cdot 2^{c_{T,1}T_{a}^{C}-c_{T,2}})$$

with

(8) $${\text{Φ}}_{CO_{2},a\_c}=(1-\exp\;(-c_{LAI,W}W))\frac{c_{I_{0}}^{phot}I_{0}(-c_{CO_{2},1}^{phot}{\left(T_{a}^{c}\right)}^{2}+c_{CO_{2},2}^{phot}T_{a}^{c}-c_{CO_{2},3}^{phot})(C_{CO_{2},a}-c_{\Gamma }^{phot})}{c_{I_{0}}^{phot}I_{0}+(-c_{CO_{2},1}^{phot}{\left(T_{a}^{c}\right)}^{2}+c_{CO_{2},2}^{phot}T_{a}^{c}-c_{CO_{2},3}^{phot})(C_{CO_{2},a}-c_{\Gamma }^{phot})}$$

Although the underlying model is parametrized with nominal values from the original authors, these estimates are typically derived from specific cultivars, production systems, and environmental regimes. Because physiological parameters in crop models vary across genotypes and production setups—including light spectra, nutrient formulations, airflow, and microclimate—using literature values without recalibration can lead to systematic prediction errors when the model is embedded in a DT. Therefore, the two parameters $c_{LAI,W}$ and $c\alpha_{\beta }$ were estimated using the parameter estimation pipeline described in Section 3.1.1 for demonstration purposes.

To this end, six growth cycles were simulated using a “true” parameter set. The sixth dataset was held out strictly for final testing. The remaining five datasets were used for 5-fold cross-validation, in which, in each fold, the parameters were estimated on four datasets and subsequently validated on the fifth. This procedure was repeated such that each of the five datasets served as the validation set exactly once. The parameter set that yielded the lowest Normalized Root Mean Square Error (NRMSE) during cross-validation was selected as the final model and evaluated against the independent test dataset. Its values are reported in **Table 1**, along with their box constraints and start values. Further, the true parameter values of this simulation model are reported. RMSE and NRMSE means across all cross-validation runs are reported in **Table 2**.

**Table 1:** Estimated lettuce model parameters start values, minimum and maximum constraints, estimated parameter and true value.

Parameter	Start	Min	Max	Estimated	True	Unit
*^c^ LAI,W*	45	22.5	90	43.79	39.5	${\text{m}}^{2}{\text{kg}}^{-1}$
*^c^αβ*	0.5	0.2500	1.0	0.4859	0.544	−

**Table 2:** Comparison of predictive performance between auto- and cross-validation.

Results	RMSE (mean)	RMSE ( σ)	NRMSE (mean)	NRMSE ( σ)
Auto validation	0.10603	0.01334	3.83%	0.48%
Cross validation	0.18065	0.12930	8.83%	4.71%

Results are reported as the mean and standard deviation (*σ*) for RMSE and NRMSE.

**Figure 4:**
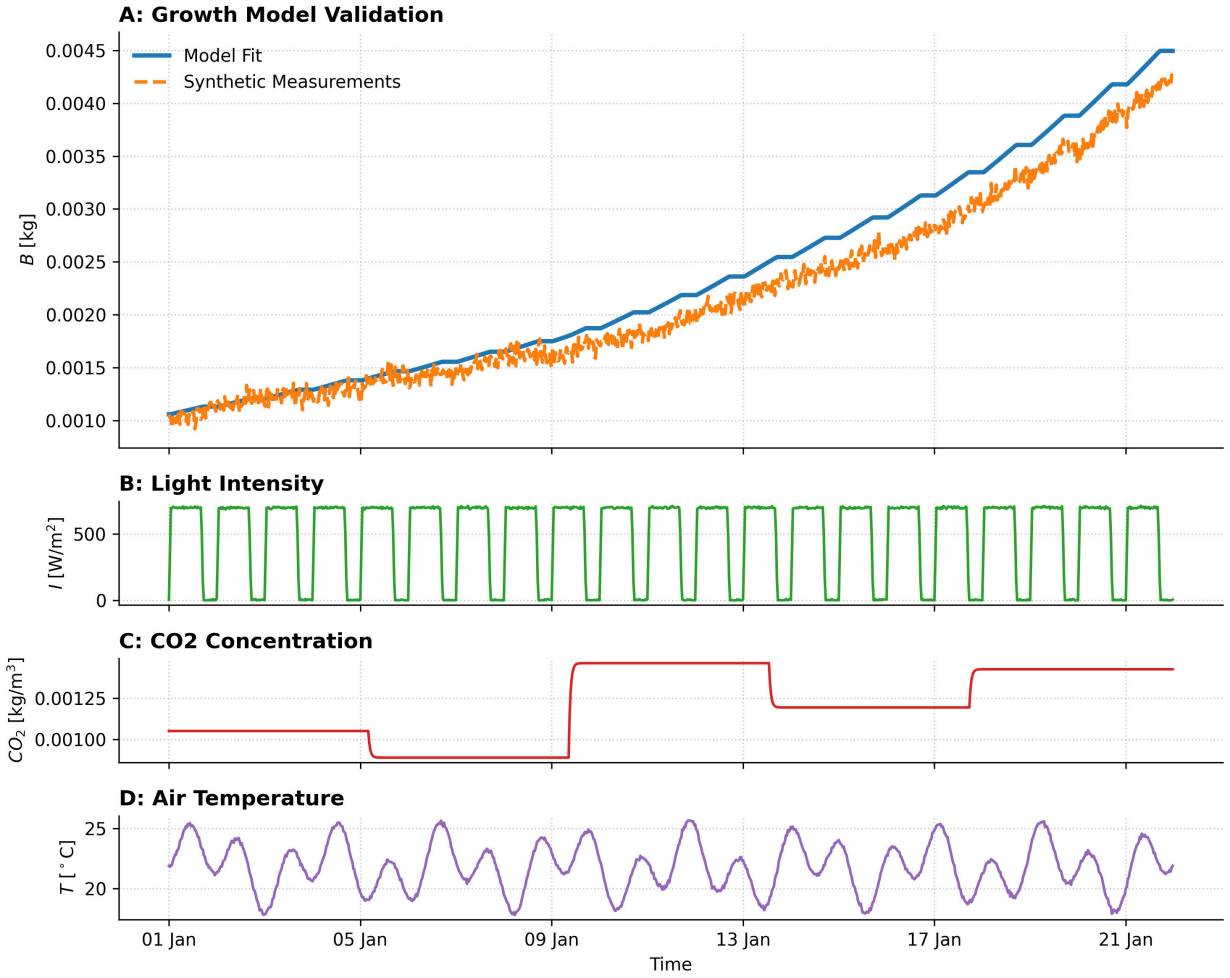
Growth model validation using synthetic test data. **(A)** Dry biomass tracking, **(B)** Light intensity input profile, **(C)** *CO*_2_ concentration input profile, and **(D)** Air temperature input profile. All trajectories and inputs shown are generated via simulation to verify the validation workflow under controlled model-mismatch conditions.

The evaluation results of the final model on the test set are presented in **Figure 4**, covering a full cultivation cycle. Specifically, **Figure 4A** illustrates the model fit of the biomass trajectory compared against the synthetic measurements, demonstrating good predictive performance with a low RMSE of 0.221g and an NRMSE of 6.62%. **Figures 4B–D** depict the corresponding time-varying environmental inputs - light intensity, *CO*_2_ concentration, and air temperature, respectively - that drive the biological growth kinetics. The slight, systematic overestimation of biomass toward the end of the growth cycle may represent a minor residual parameter identifiability artifact, where the model structure marginally overpredicts late-stage kinetic rates under quasi-static conditions.

It is important to note that the thermodynamic behavior of the production unit (e.g., HVAC dynamics, heat transfer through the climate tent walls, and thermal loads introduced by the LEDs) is not explicitly modeled in this case study. Instead, the microclimate variables driving the biological growth model are treated as directly measured environmental inputs. While a comprehensive, facility-scale DT requires coupling these multi-domain thermodynamic interactions to achieve holistic resource optimization, such physical infrastructure modeling falls outside the scope of the present work.

#### 4.1.2 Multi-rate Moving Horizon Estimation for biomass

In the case study the DT’s MMHE service was employed to address lettuce growth monitoring with infrequent measurement data. It was assumed that dry biomass was measured every 4.75 days by manually weighing individual lettuce specimens and attributing 5% of their fresh biomass to their dry biomass content. The estimator was configured with a sampling period of Δ*t* = 30 minutes and a horizon length of *N* = 5 steps. Thus, the MMHE’s horizon was significantly smaller than the interval between incoming measurements, meaning that the estimator operates predominantly in prediction mode. In this state, the DT relies on the underlying mechanistic model and occasionally applies corrections when new biomass measurement data become available.

The MMHE was tested on a simulated lettuce growth cycle constructed using synthetic dry-biomass measurements. These measurements were generated by forward simulation of the “true” lettuce model. To evaluate the estimator’s robustness and its ability to handle the inherent uncertainty of biological systems as well as of the measurement equipment, the simulation was subjected to both additive Gaussian measurement and process noise with a standard deviation of 0.05g and 0.006g each. The MMHE was configured with the previously fitted growth model. This created an intentional process-model mismatch to simulate the discrepancy between a simplified mathematical representation and complex biological reality.

The weighting matrices Q and R*^S^*, which represent the inverse covariances of the process noise and the slow (intermittent) measurement noise, respectively, were both scalar in this case study. They were manually tuned to balance model fidelity against the magnitude of measurement corrections at Q = 1*e* – 6 and R*^S^* = 1*e* − 5.

The MMHE was initialized at time *t* = *t*_0_ + *N*Δ*t* to ensure a full data buffer was available for the first optimization window. We assumed that the biomass measurement was recorded at the initial time *t*_0_, and the initial state estimate ${\hat{W}}_{0}$ was set equal to this measurement.

**Figure 5:**
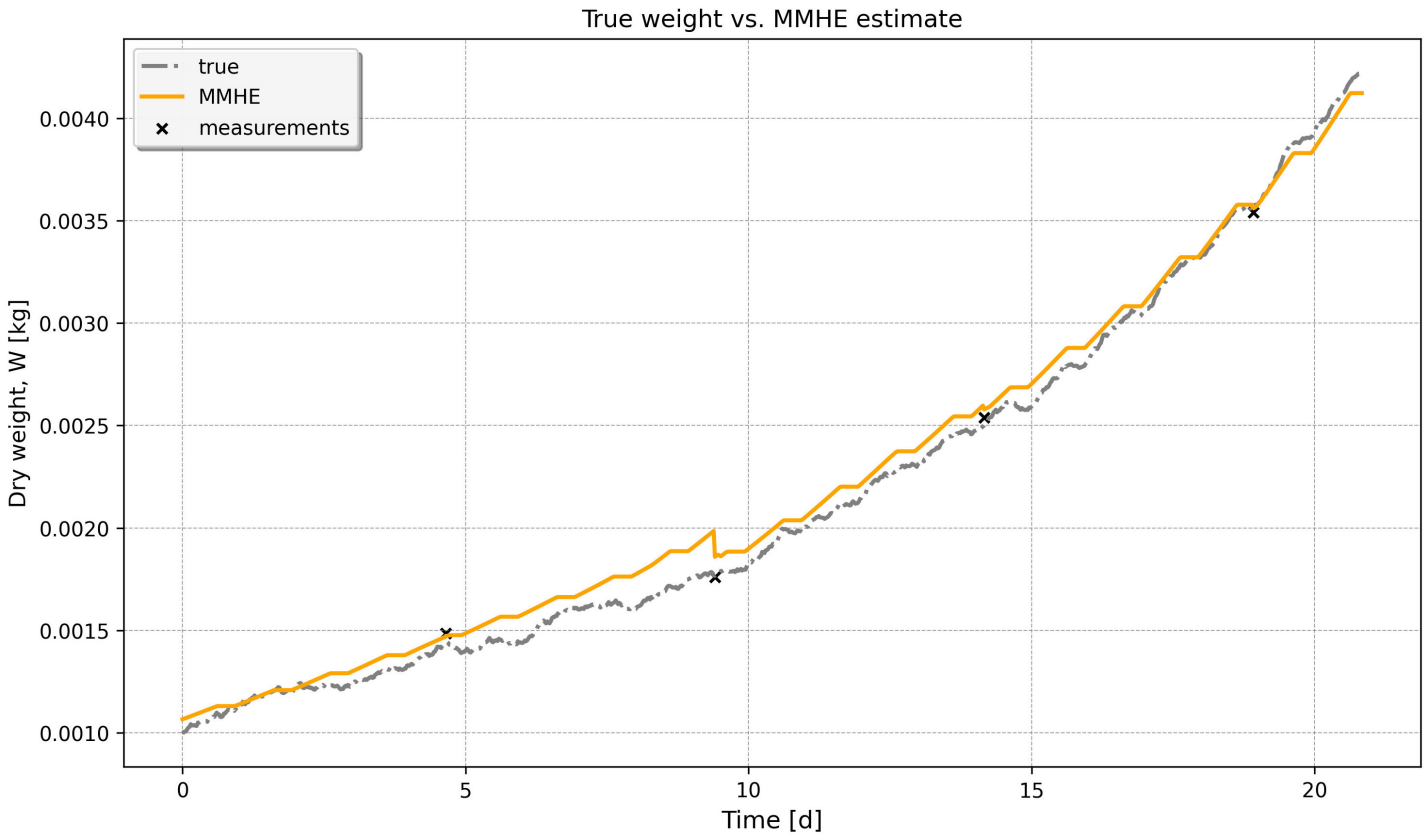
MMHE state estimation of lettuce dry weight compared to true simulated values. The discrete jumps in the orange estimate occur upon the arrival of new measurements, providing periodic corrections to the simulation’s trend and ensuring the long-term accuracy of the DT’s biomass prediction.

As illustrated in **Figure 5**, the MMHE initially overestimated the lettuce growth slightly, especially between the measurements from days 4 and 9. The measurement update at day 9 corrected the estimated biomass trajectory toward the true trajectory. For the remaining 12 days, biomass estimation and the true trajectory showed good alignment, with only minor measurement-update corrections. Because the estimator and the synthetic ground truth share the same model structure, the state corrections at measurement times are expectedly modest. Larger corrections would be anticipated to occur in real-world deployments facing structural model mismatches.

#### 4.1.3 Human-machine interface

**Figure 6:**
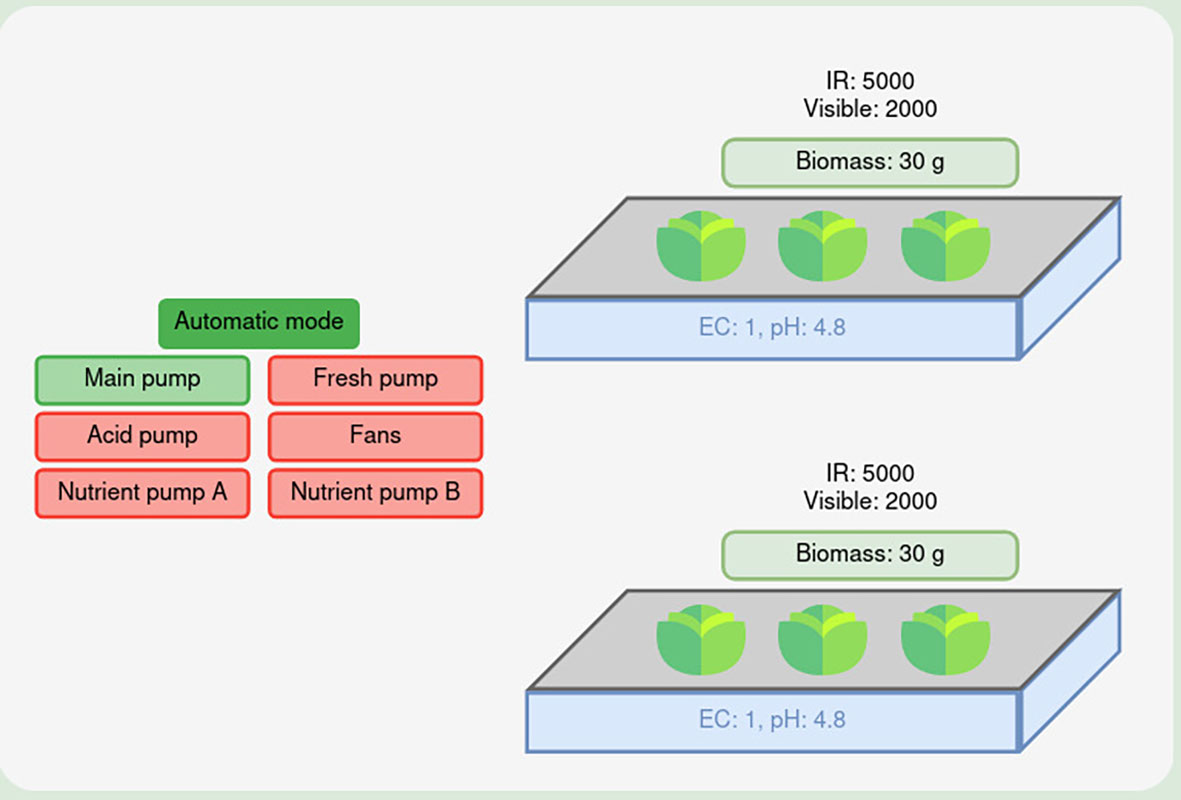
GreenResearcher SCADA widget.

An HMI was developed via multiple dashboards in ThingsBoard. This included a timeseries widget to display the production unit’s thermodynamic properties temperature, humidity and CO2 over the last 24 hours. Further, a SCADA-like widget shown in **Figure 6** was developed and deployed. It provides an interactive visualization of the GR unit and its current operational state, based on data streamed from its embedded and auxiliary sensors, as well as biomass estimates from the MMHE. The interface displays key environmental and system variables and enables direct user manipulation of selected actuators, such as pumps and ventilation units, via button-based controls that communicate with the corresponding RPC handlers to command actuation.

**Figure 7:**
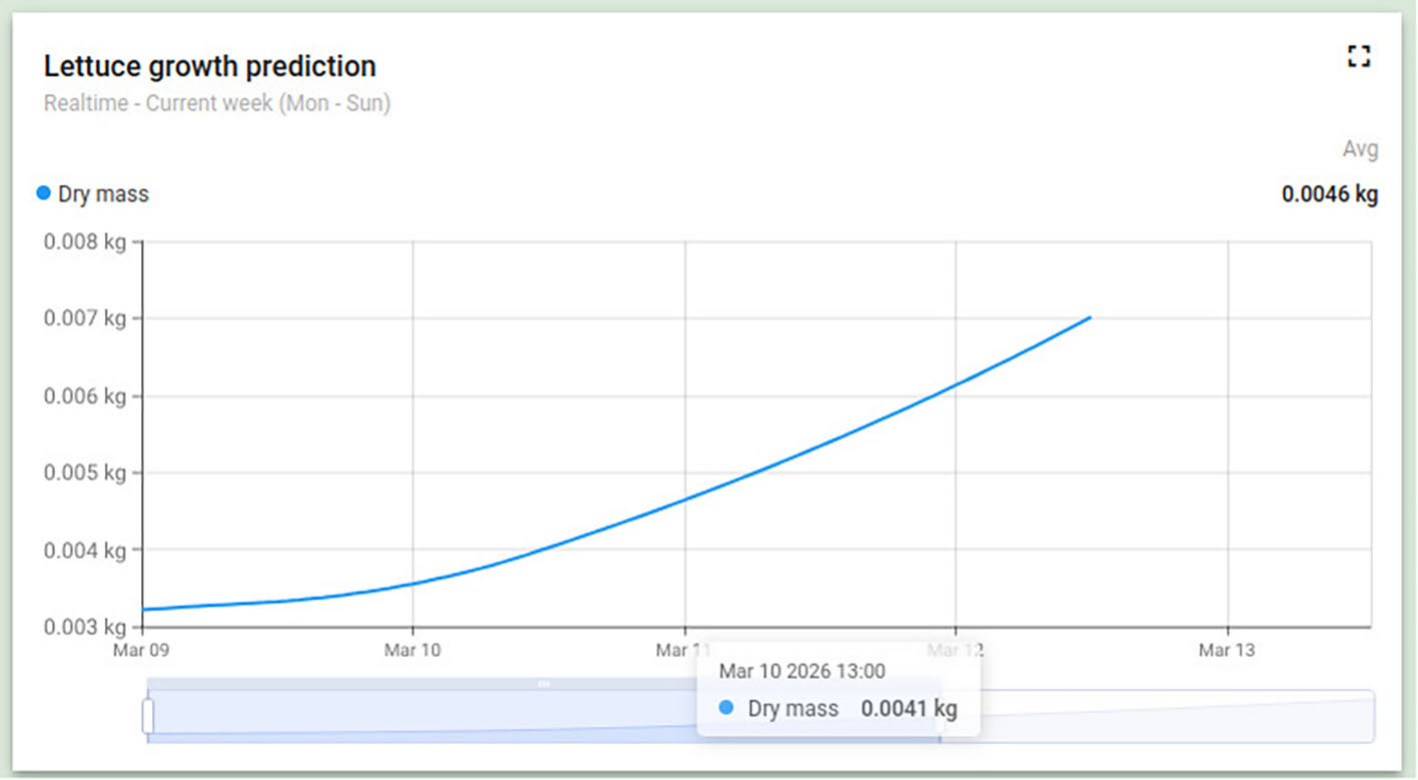
Lettuce dry biomass growth prediction shown on HMI.

Further, the model simulation backend was set up to predict the biomass growth at time steps of one hour for the next 3.5 days. Predictions were displayed in a ThingsBoard “time series chart” widget as shown in **Figure 7**.

#### 4.1.4 Computational performance metrics

To evaluate the feasibility of the proposed framework for real-time applications, execution and cycle times were measured during the case study. The MMHE pipeline demonstrated exceptional optimization speeds, achieving approximately 99.28 iterations per second. The predictive simulation backend also proved highly efficient; the physical simulation execution itself occurred near-instantaneously. The end-to-end simulation cycle time—encompassing the downloading of physical measurements from the database, model execution, and the uploading of simulation outputs—consistently remained in the vicinity of 1s.

## 5 Discussion

Although this study demonstrates the framework using a hydroponic lettuce case study, the architecture is, in principle, crop-agnostic, given a suitable ODE-based model integrated via the FMI standard. Extending this framework to fruiting crops, such as tomato or strawberry, would necessitate multi-state models that account for longer growth cycles and additional sensing requirements, such as fruit load and biomass partitioning. However, open-field crops fall outside this demonstrated scope, as they lack the continuous sensing and controlled climate necessary for our current framework.

Our framework provides a concrete operationalization of the Process-Based Model (PBM) centered digital twin architecture recently discussed by Rao et al. (2026). While Rao et al. (2026) emphasize that the deployment of such twins depends heavily on resolving technical hurdles related to data integration and infrastructural demands, our architecture addresses these by utilizing a modular, cloud-native stack.

Specifically, **Figure 2** illustrates how the central IoT platform ThingsBoard is utilized to manage real-time data acquisition. Services such as state estimators and controllers are currently implemented as modular, independent services, yet future deployment via containerization technologies like Docker could further streamline operations. By isolating environments, such an approach would simplify scaling and prevent conflicting software dependencies, bridging the gap between process-based modeling and operational management—a key challenge identified in current literature.

The presented DT framework directly addresses critical issues in existing CEA DT implementations, such as scalability, interoperability, and the seamless integration of predictive models with live data streams (Monteiro et al., 2023). In contrast to monitoring-centric approaches that primarily focus on data aggregation and visualization, this framework elevates predictive simulation and state estimation to dedicated DT services, thereby enabling predictive functionality and paving the way for advanced control. This is of particular importance in the context of CEA, where critical variables such as crop growth states are frequently unmeasurable. Consequently, the requirement is for continuously updated internal state representations rather than mere sensor data visualization. A fundamental element of the framework’s robustness is its modular, open-source design, which leverages the IoT platform ThingsBoard as the core integration layer for unified device integration and user interaction. This choice is supported by comparative analyses that highlight ThingsBoard’s maturity and rich features in device management and visualization.

The framework’s capacity to aggregate communication protocols and data formats into a consolidated representation effectively decouples hardware from software, thereby ensuring architectural scalability and flexibility without vendor lock-in. The DT framework is predicated on the utilization of dynamic ODE models, developed in Modelica, to capture complex biological and thermodynamic interactions in CEA environments. A significant contribution is the use of FMUs via the FMI standard, which ensures model interoperability and reusability across various simulation, parameter estimation, and control services. The decoupling of model formulation from execution is crucial for integrating diverse modeling tools without extensive redesign.

The integration of developed grey-box models into the DT was facilitated by a dedicated parameter estimation pipeline that employed MLE. The pipeline automates the calibration of FMU-based models against experimental data, thereby addressing the challenge of varying physiological parameters across different crop cultivars and facility architectures. Practical challenges posed by heterogeneous, sparse, and non-uniformly sampled sensor data are also addressed. Currently, the pipeline requires manual initiation for parameter recalibration, followed by a manual override of the updated parameters within the respective MMHE and simulation backend files. Transitioning this process to a fully automated, closed-loop update cycle remains an objective for future development. The case study demonstrated the pipeline’s effectiveness, with the calibrated growth model achieving a low cross-validation prediction error (RMSE of 0.221g and NRMSE of 6.62% on an independent test set), confirming its ability to generalize beyond fitting data. To contextualize these figures against the literature, our framework achieves a Mean Absolute Percentage Error (MAPE) of 8.24% across the entire independent test set without any outlier filtering. In comparison, earlier calibrations of the more flexible two-state van Henten model reported tracking errors mostly within 2% to 3% but did not account for operational outliers (Abedi et al., 2023). Furthermore, our test-set NRMSE of 6.62% compares favorably to the three-compartment mechanistic lettuce model by Rohde and Forni (2023), which achieved fitting NRMSE values ranging between 3% and 11% under highly controlled indoor conditions. Finally, while this core architecture is inherently designed for a bidirectional Digital Twin via integrated RPC handlers, the current case study implementation did not include automated control logic for the GreenResearcher’s actuators. Consequently, pending the deployment of controllers (e.g., using MPC), the present implementation serves as a control-ready Digital Shadow.

A critical innovation is the integration of an MMHE state estimator, which acts as a soft-sensor service to estimate unmeasured variables such as plant biomass under sparse measurement regimes. This capability is vital for advanced dynamic control schemes and anomaly detection. To ensure the evaluation remains representative of typical commercial CEA facilities—where direct, continuous biomass weight sensors are economically or operationally impractical—the MMHE framework was validated in silico using a synthetic dataset rather than physical weight sensors. Within this simulated environment, the estimator demonstrated promising performance and resilience under a controlled parameter mismatch. It should be noted that in practical CEA applications, structural mismatch—where the real plant exhibits dynamics not captured by the underlying equations—presents a more significant challenge, requiring dedicated mitigation methods. Further, the weighting matrices (*Q* and *R^S^*) were tuned manually to balance model confidence against measurement noise, and validating this MMHE framework against real-world crop growth dynamics remains an objective for future work. A major implication of manual tuning is its dependency on human intuition, which can cause tracking performance to deteriorate if real-world noise characteristics shift. In industrial or commercial CEA applications, these tuning parameters could instead be selected systematically using covariance matching techniques, autocovariance least-squares methods, or optimization routines calibrated against historical operational datasets.

While an offline-calibrated PBM may perform well under the static, controlled set-points of our short-term case study, its true value diminishes in complex, dynamic production environments. Over longer cycles, open-loop simulations inevitably drift due to unmodeled biological changes or sensor degradation. Furthermore, in commercial vertical farms, microclimatic conditions like air temperature vary significantly from layer to layer and cannot be measured everywhere. Because individual trays experience localized environmental variations, a static model becomes inaccurate. The integrated IoT layer addresses this by continuously gathering multi-rate measurements and coordinating real-time actuation via RPC commands. Crucially, the MMHE layer leverages these sparse measurements to continuously correct the state estimation, ensuring tracking fidelity under both localized disturbances and dynamic operating conditions like energy-aware climate control.

Furthermore, transferring this online estimator to physical production units requires a clear understanding of its performance in the presence of non-ideal data anomalies. The MMHE formulation provides an inherent structural advantage when handling data loss and communication delays (Elsheikh et al., 2021). Specifically, timepoints at which data loss occurs can simply be excluded from the optimization window by leveraging the sparse selection mapping matrix, preventing missing data from destabilizing the objective function. Similarly, communication delays do not compromise tracking accuracy as long as the network lag is shorter than the estimation horizon length. Since incoming measurements are timestamped at the source, late-arriving data can be retrospectively placed into its correct chronological position and re-solved within the moving window. Conversely, persistent problems such as sensor drift or extreme outliers would degrade tracking accuracy if left unaddressed. In practical industrial deployments, these vulnerabilities must be mitigated, for example, by implementing outlier rejection filters at the IoT middleware layer before data reaches the estimator.

In terms of practical implementation, the framework employs MDS for continuous, asynchronous data collection from sensors and RPC handlers for relaying actuation commands to physical actuators. This vendor-agnostic approach, facilitated by ThingsBoard, ensures flexible integration of heterogeneous sensor types and actuators, which is crucial for scalability in and interoperability across diverse CEA environments. The utilization of interactive dashboards in ThingsBoard streamlines user interaction, providing comprehensive monitoring, status indicators, and SCADA-style visualizations. This HMI enables manual control of actuators and visualizes predictions from the simulation backend, thereby making DT’s insights actionable for operators.

The proposed DT framework is designed to be scalable, potentially enabling its deployment across large-scale facilities with multiple production units. However, empirical validation of this scalability remains a subject for future research. By leveraging a central server, multiple MMHE instances and controllers can be executed in parallel, each dedicated to a specific production unit. This parallel execution is further supported by the predictive simulation backend, which utilizes an asynchronous design to efficiently manage and simulate a large volume of systems simultaneously. Furthermore, the architecture imposes no inherent limitations on the number of networked devices, enabling the system to expand incrementally over time through its modular nature. The framework’s flexibility is enhanced by the use of various communication protocols within the MDS and RPC handlers, which bridge the IoT platform to vendor-specific protocols. This ensures that new sensors and actuators from diverse vendors can be integrated seamlessly into the growing ecosystem without requiring core architectural changes.

To bridge the gap between architectural capability and industrial deployment, several practical cyber-physical constraints and computational costs must be considered. As demonstrated by the computational performance metrics in Section 4.1.4, both the MMHE optimization loop and the predictive simulation backend exhibit substantial computational headroom. When combined with the framework’s asynchronous design and the isolated parallelization of individual state estimators, these performance margins ensure sufficient capacity for real-time operation across multiple concurrent nodes. Regarding network topologies, while communication latency between a centralized server and network devices could affect upper-level optimizations, safety-critical local control loops are intended to operate independently on their respective devices (such as a PLC). Thus, minor network lags will not critically destabilize physical operations. Furthermore, when addressing potential time synchronization challenges, a clear distinction must be made between sensing and actuation: because all incoming sensor measurements are strictly timestamped at the source, data loss or network delays can be handled seamlessly by the database and the retrospective re-solving capability of the MMHE service. However, because outgoing actuation commands sent via RPC handlers are not timestamped, high-frequency control actions, such as actuator chattering, must be explicitly mitigated within the upper-level controller design to prevent network jitter issues. Finally, moving to a commercial multi-zone facility will require a detailed evaluation of server computational overhead from running concurrent predictive simulations and state estimations alongside rigorous end-to-end security hardening to protect proprietary operational data.

Interoperability is a fundamental principle of the framework’s design, primarily realized through the standardized integration of FMUs. The framework is characterized by a high degree of modularity, which is ensured by the embedding of FMUs into the parameter estimation pipeline, the MMHE, and the predictive simulation backend. This approach ensures that the DT remains tool-agnostic, permitting models to be developed in various software environments, such as Python, MATLAB, or Modelica, and then seamlessly incorporated into the CEA workflow. Furthermore, it has been demonstrated that the same structural model of a production unit or biological process can be utilized across different CEA facilities and calibrated via the parameter estimation pipeline prior to deployment.

Despite the robust design of the framework, there are certain limitations and key areas for future work (Monteiro et al., 2023). While the ODE-based modeling workflow was successfully demonstrated in the case study, only a simple single-state lettuce model has been implemented to date, and the thermodynamics of the production system have not been modeled. This level of simplification was appropriate for the primary objective of this study, which was to validate the underlying software architecture, FMI interoper-ability, and multirate data-ingestion pipelines rather than introducing a novel biological model. However, neglecting key biological processes such as *CO*_2_ consumption and transpiration limits the strength of our conclusions regarding immediate applicability for complex dynamic control, resource optimization, and advanced plant physiological analysis in CEA. As noted by Padmanabha et al. (2023), a comprehensive CEA model must account for the bidirectional feedback between the physical infrastructure and the cultivated species. Because our current case study does not capture the production unit’s thermodynamic behavior, it does not constitute a complete or high-fidelity biological DT. Without an explicit thermodynamic infrastructure model, direct resource-aware optimizations—such as minimizing HVAC energy consumption relative to dynamic electricity pricing—cannot be demonstrated. Consequently, the present validation remains focused on architectural integration and biomass prediction rather than the complete optimization of the physical production environment. To move beyond these boundaries, subsequent research will focus on incorporating an open-source, multi-physical modeling library into this DT framework to natively support multi-domain coupling. Transitioning to these more complex models may introduce challenges related to parameter identifiability, state observability, and computational demands, which must be systematically addressed. Furthermore, the integration of data-driven or hybrid models represents a promising extension of this framework, as they simplify parameterization while maintaining high tracking accuracy in real-time applications (Fan et al., 2015; Rao et al., 2026).

The modular DT framework represents a significant advancement for CEA, offering a robust, scalable, and interoperable solution that was validated at an advanced technology readiness level in a laboratory-scale case study. However, further evaluation is required to generalize its application to multi-zone, large-scale CEA production facilities, particularly regarding network robustness, time synchronization, and security hardening. The case study excluded the thermodynamic behavior of the production unit, assuming direct measurement of environmental inputs; nevertheless, the framework is explicitly designed to enable integration of thermodynamic models with biological dynamics, representing a rational progression toward a comprehensive facility-scale DT capable of balancing energy efficiency, maximizing biomass, and optimizing actuator performance. The emphasis on physics-based modeling, advanced state estimation techniques, and an open-source IoT architecture provides a strong foundation for resource-efficient and energy-aware operations in complex CEA environments. Subsequent research will therefore need to concentrate on implementing MPC using states estimated by state estimation techniques, in conjunction with the systematic evaluation of the DT framework against formal maturity criteria and service taxonomies (Brentarolli, 2022; Soussi et al., 2025). This will further substantiate its practical utility and broaden its applicability across diverse CEA scenarios.

## 6 Conclusion

This study successfully introduced a novel open-source IoT-enabled DT framework for vertical farming, specifically designed to target common limitations in existing DT implementations in CEA, such as scalability, interoperability, and the seamless integration of predictive models with live data streams. The IoT platform ThingsBoard is positioned as the central integration layer, thereby ensuring unified device integration and user interaction. This framework is supported by modular backend services for state trajectory prediction, state estimation, and control. A fundamental contribution of the framework is its physics-based modeling backend, which utilizes Modelica and FMUs via the FMI standard to enable real-time state estimation and predictive simulation. Crucially, numerical simulations demonstrated the potential of the integrated MMHE state estimator to continuously estimate critical unmeasured variables, such as plant biomass, even under conditions of infrequent measurements and process-model incompatibility. This capability ensures the maintenance of operationally useful state estimates within the DT which could be used for decision making processes and advanced control algorithms in the future. However, because these validation steps, including the parameter estimation pipeline, relied on synthetic datasets and a simplified single-state biological model, complete physical validation remains an open objective. Future work will focus on transitioning the framework to a live facility to capture production unit thermodynamics, integrate multi-state crop models, and empirically test the framework’s scalability in a large-scale commercial environment. Ultimately, this framework signifies a considerable architectural advancement toward dynamic, data-driven, and energy-aware operations in CEA, establishing a robust and extensible foundation for advanced control strategies such as MPC and comprehensive facility DTs.

## Data Availability

The original contributions presented in the study are included in the article/**Supplementary Material**. Further inquiries can be directed to the corresponding author.

## Supplementary material

The Supplementary Material for this article can be found onlineat: https://www.frontiersin.org/articles/10.3389/fpls.2026.1864757/full#supplementary-material

## Appendix Case study simulation backend configuration

The following YAML file was used to configure the predictive simulation backend to use the lettuce model. This file defines solvers, state synchronization parameters, and unit conversion factors.Listing 1. YAML configuration for the Predictive Simulation Backend.

general:

fmu_name: “Lettuce.fmu”

step_size: 3600

num_t_steps: 48

solver: “cvodes”

sim_device_token: “8LMkgr6hbkh7oeeeS8P7”

simulation_interval: 10.0 *# seconds*

input_mode: “constant” *# “constant” or “trajectory”*

*# 1. Global Parameters (Static during simulation)* parameters:

- name: “k_LAI”

value: 53

- name: “k_a_med”

value: 0.12

*# 2. State Mapping (Feedback loop from the real world)* states:

- tb_var: “Totalweight”

fmu_var: “lettuce.B”

device_token: “place_holder_token_a”

device_name: “GreenResearcher_1”

prediction_var_name: “B”

conversion_factor: 5e-3

*# 3. Inputs Mapping* inputs:

- tb_name: “scd4xCo2”

fmu_var: “C”

device_token: “place_holder_token_b”

device_name: “Arduino_1”

default_value: 400

conversion_factor: 1.799e-6

- tb_name: “bme280T”

fmu_var: “T”

device_token: “place_holder_token_b”

device_name: “Arduino_1”

default_value: 24

conversion_factor: 1

- tb_name: “ppfd”

fmu_var: “I”

device_token: “place_holder_token_b”

device_name: “Arduino_1”

default_value: 400

conversion_factor: 1

*# 4. Outputs*

outputs: []

## References

[B2] Abedi M. Tan X. Stallknecht E. J. Runkle E. S. Klausner J. F. Murillo M. S. . (2023). Incorporating the effect of the photon spectrum on biomass accumulation of lettuce using a dynamic growth model. Front. Plant Sci. 14. doi: 10.3389/fpls.2023.1106576 37360721 PMC10286798

[B3] Andersson J. (2024). “ Import and export of functional mockup units in casadi”, in: Proceedings of the 15th International Modelica Conference 2023, Aachen, October 9-11 ( Linköping University Electronic Press), 204, 321–326. doi: 10.3384/ecp204321

[B4] Andersson J. A. E. Gillis J. Horn G. Rawlings J. B. Diehl M. (2018). Casadi: a software framework for nonlinear optimization and optimal control. Math. Program. Comput. 11, 1–36. doi: 10.1007/s12532-018-0139-4 30311153

[B5] Ariesen-Verschuur N. Verdouw C. Tekinerdogan B. (2022). Digital twins in greenhouse horticulture: A review. Comput. Electron. Agric. 199, 107183. doi: 10.1016/j.compag.2022.107183 38826717

[B6] Arshi O. Mondal S. (2023). Advancements in sensors and actuators technologies for smart cities: a comprehensive review. Smart Construction Sustain. Cities 1(1), 18. doi: 10.1007/s44268-023-00022-2 30311153

[B7] Beacham A. M. Vickers L. H. Monaghan J. M. (2019). Vertical farming: a summary of approaches to growing skywards. J. Hortic. Sci. Biotechnol. 94, 277–283. doi: 10.1080/14620316.2019.1574214 37339054

[B8] Boudreau M. A. McMillan G. K. (2007). New Directions in Bioprocess Modeling and Control: Maximizing Process Analytical Technology Benefits (North Carolina: ISA).

[B9] Brentarolli E. (2022). Towards A Digital Twin for Agriculture: Modeling of Complex Processes for Monitoring, Prediction and Control in Greenhouse Farming. Università degli Studi di Verona, Verona, Italy.

[B10] Cañas C. D. Arroyo J. Gillis J. Helsen L. (2023). “ Parameter estimation of modelica building models using casadi”, in: Proceedings of the 15th International Modelica Conference 2023, Aachen, October 9-11 ( Linköping University Electronic Press), 204, 301–310. doi: 10.3384/ecp204301

[B11] Chaux J. D. Sanchez-Londono D. Barbieri G. (2021). A digital twin architecture to optimize productivity within controlled environment agriculture. Appl. Sci. 11, 8875. doi: 10.3390/app11198875 30654563

[B12] Classens K. Heemels W. M. Oomen T. (2021). “ Digital twins in mechatronics: From model-based control to predictive maintenance”, in: 2021 IEEE 1st International Conference on Digital Twins and Parallel Intelligence (DTPI) (Beijing: IEEE), 336–339. doi: 10.1109/dtpi52967.2021.9540144

[B13] David I. Shao G. Gomes C. Tilbury D. Zarkout B. (2024). “ Interoperability of digital twins: Challenges, success factors, and future research directions”, in: Leveraging Applications of Formal Methods, Verification and Validation. Application Areas ( Springer Nature Switzerland), 27–46. doi: 10.1007/978-3-031-75390-9_3

[B14] De Nardis L. Mohammadpour A. Caso G. Ali U. Di Benedetto M.-G. (2022). Internet of things platforms for academic research and development: A critical review. Appl. Sci. 12, 2172. doi: 10.3390/app12042172 30654563

[B15] Di Felice P. (2023). “ A systematic mapping study about iot platforms”, in: The 4th International Electronic Conference on Applied Sciences (Basel: MDPI). 226. doi: 10.3390/asec2023-15388

[B16] Elsheikh M. Hille R. Tatulea-Codrean A. Krämer S. (2021). A comparative review of multi-rate moving horizon estimation schemes for bioprocess applications. Comput. Chem. Eng. 146, 107219. doi: 10.1016/j.compchemeng.2020.107219 38826717

[B17] Falcão R. Matar R. Rauch B. (2022). “ Using i4. 0 digital twins in agriculture”, in: European Conference on Software Architecture (Cham: Springer), 483–498.

[B18] Fan X.-R. Kang M.-Z. Heuvelink E. de Reffye P. Hu B.-G. (2015). A knowledge-and-data-driven modeling approach for simulating plant growth: A case study on tomato growth. Ecol. Modell. 312, 363–373. doi: 10.1016/j.ecolmodel.2015.06.006 38826717

[B19] Fritzson P. Pop A. Abdelhak K. Ashgar A. Bachmann B. Braun W. . (2020). The open-modelica integrated environment for modeling, simulation, and model-based development. Modeling Identification Control: A. Norwegian Res. Bull. 41, 241–295. doi: 10.4173/mic.2020.4.1

[B20] González J. P. Sanchez-Londoño D. Barbieri G. (2022). A monitoring digital twin for services of controlled environment agriculture. IFAC-PapersOnLine 55, 85–90. doi: 10.1016/j.ifacol.2022.09.188 38826717

[B21] Graamans L. Baeza E. van den Dobbelsteen A. Tsafaras I. Stanghellini C. (2018). Plant factories versus greenhouses: Comparison of resource use efficiency. Agric. Syst. 160, 31–43. doi: 10.1016/j.agsy.2017.11.003 38826717

[B22] Grieves M. Vickers J. (2017). “ Digital twin: mitigating unpredictable, undesirable emergent behaviour in complex systems,” in Transdisciplinary perspectives on complex systems: New findings and approaches (Cham: Springer International Publishing, Switzerland), 85–113.

[B23] Gund R. Badgujar C. M. Samiappan S. Jagadamma S. (2025). Application of digital twin technology in smart agriculture: A bibliometric review. Agriculture 15, 1799. doi: 10.20944/preprints202507.1502.v1

[B24] Ismail A. A. Hamza H. S. Kotb A. M. (2018). “ Performance evaluation of open source iot platforms”, in: 2018 IEEE Global Conference on Internet of Things (GCIoT) (Alexandria: IEEE), 1–5. doi: 10.1109/gciot.2018.8620130

[B25] Javaid M. Haleem A. Suman R. (2023). Digital twin applications toward industry 4.0: A review. Cognit. Rob. 3, 71–92. doi: 10.1016/j.cogr.2023.04.003 38826717

[B26] Jones D. Snider C. Nassehi A. Yon J. Hicks B. (2020). Characterising the digital twin: A systematic literature review. CIRP J. Manuf. Sci. Technol. 29, 36–52. doi: 10.1016/j.cirpj.2020.02.002 38826717

[B27] Kabir M. S. N. Reza M. N. Chowdhury M. Ali M. Samsuzzaman Ali M. R. . (2023). Technological trends and engineering issues on vertical farms: A review. Horticulturae 9, 1229. doi: 10.3390/horticulturae9111229 30654563

[B28] Kaiser E. Kusuma P. Vialet-Chabrand S. Folta K. Liu Y. Poorter H. . (2024). Vertical farming goes dynamic: optimizing resource use efficiency, product quality, and energy costs. Front. Sci. 2. doi: 10.3389/fsci.2024.1411259

[B29] Kalantari F. Tahir O. M. Joni R. A. Fatemi E. (2018). Opportunities and challenges in sustainability of vertical farming: A review. J. Landscape Ecol. 11, 35–60. doi: 10.1515/jlecol-2017-0016 31755547

[B30] Kozai T. (2013). Resource use efficiency of closed plant production system with artificial light: Concept, estimation and application to plant factory. Proc. Japan Academy Ser. B. 89, 447–461. doi: 10.2183/pjab.89.447 24334509 PMC3881955

[B32] Krämer S. Gesthuisen R. (2005). Multirate state estimation using moving horizon estimation. IFAC Proc. Volumes 38, 1–6. doi: 10.3182/20050703-6-cz-1902.00654

[B31] Kritzinger W. Karner M. Traar G. Henjes J. Sihn W. (2018). Digital twin in manufacturing: A categorical literature review and classification. IFAC-PapersOnLine 51, 1016–1022. doi: 10.1016/j.ifacol.2018.08.474 38826717

[B33] Marvin S. Rutherford J. (2018). Controlled environments: An urban research agenda on microclimatic enclosure. Urban Stud. 55, 1143–1162. doi: 10.1177/0042098018758909

[B34] Monteiro J. Barata J. Veloso M. Veloso L. Nunes J. (2018). “ Towards sustainable digital twins for vertical farming”, in: 2018 Thirteenth International Conference on Digital Information Management (ICDIM) (Berlin: IEEE), 234–239. doi: 10.1109/icdim.2018.8847169

[B35] Monteiro J. Barata J. Veloso M. Veloso L. Nunes J. (2023). A scalable digital twin for vertical farming. J. Ambient Intell. Hum. Comput. 14, 13981–13996. doi: 10.1007/s12652-022-04106-2 30311153

[B36] O’Connell E. O’Brien W. Bhattacharya M. Moore D. Penica M. (2023). Digital twins: Enabling interoperability in smart manufacturing networks. Telecom 4, 265–278. doi: 10.3390/telecom4020016 30654563

[B37] Padmanabha M. Kobelski A. Hempel A.-J. Streif S. (2023). Modelling and optimal control of growth, energy, and resource dynamics of hermetia illucens in mass production environment. Comput. Electron. Agric. 206, 107649. doi: 10.1016/j.compag.2023.107649 38826717

[B38] Rao S. Ahmadi S. H. Leitner D. Vianna M. Bauer F. M. Seidel S. J. . (2026). Integrated modeling approaches for agricultural digital twins: the role of process based models, agent based models, machine learning, and model coupling. In Silico Plants 8 (1). doi: 10.1093/insilicoplants/diag002 40388063

[B39] Renard D. Saddem R. Annebicque D. Riera B. (2024). From sensors to digital twins toward an iterative approach for existing manufacturing systems. Sensors 24, 1434. doi: 10.3390/s24051434 38474970 PMC10934656

[B40] Rohde W. Forni F. (2023). Lettuce modelling for growth control in precision agriculture. Eur. J. Control 74, 100843. doi: 10.1016/j.ejcon.2023.100843 38826717

[B1] Shittu M. A. Shittu H. A. Adeleke O. J. Adedokun O. J. (2023). Digital twin modeling for real time monitoring and fault detection in smart substations. Int. J. Ind. Eng. Res. Dev. 14, 25–44. doi: 10.34218/ijierd_14_02_003 30362018

[B41] Soussi A. Zero E. Herrera C. D. C. Zahmoun S. Bozzi A. Sacile R. (2025). Integrating digital twins and mpc for sustainable greenhouse management in smart agriculture. IEEE Trans. Agrifood Electron. 4(1), 39–55. doi: 10.1109/tafe.2025.3572808 25079929

[B42] Tao F. Qi Q. Liu A. Kusiak A. (2018). Data-driven smart manufacturing. J. Manuf. Syst. 48, 157–169. doi: 10.1016/j.jmsy.2018.01.006 38826717

[B43] Tekinerdogan B. Verdouw C. (2020). Systems architecture design pattern catalog for developing digital twins. Sensors 20, 5103. doi: 10.3390/s20185103 32906851 PMC7570903

[B44] ThingsBoard Authors (2026). Thingsboard: Open-source iot platform.

[B45] ThingsBoard Team (2026a). ThingsBoard Python Client SDK. Available online at: https://github.com/thingsboard/thingsboard-python-client-sdk (Accessed April 24, 2026).

[B46] ThingsBoard Team (2026b). ThingsBoard Python REST API Client. Available online at: https://github.com/thingsboard/thingsboard-python-rest-client (Accessed April 24, 2026).

[B47] Turki M. El Boussaidi G. Benzarti I. Mili H. (2024). “ Evaluating open source iot platforms: A github analysis”, in: Proceedings of the ACM/IEEE 6th International Workshop on Software Engineering Research & Practices for the Internet of Things (Lisbon: ACM), 14–21. doi: 10.1145/3643794.3648348

[B48] van Henten E. (1994). Greenhouse Climate Management: An Optimal Control Approach (The Netherlands: Wageningen University and Research).

[B49] Verdouw C. Tekinerdogan B. Beulens A. Wolfert S. (2021). Digital twins in smart farming. Agric. Syst. 189, 103046. doi: 10.1016/j.agsy.2020.103046 38826717

[B50] Wächter A. Biegler L. T. (2006). On the implementation of a primal-dual interior point filter line search algorithm for large-scale nonlinear programming. Math. Program. 106, 25–57. doi: 10.1007/s10107-004-0559-y 30311153

