# Supplementary figures and images for: Dynamic, adaptive and modular Digital Twin framework for resource-efficient Controlled Environment Agriculture
Source: Front Plant Sci. 2026 Jul 1;17:1864757. doi: 10.3389/fpls.2026.1864757 (PMC13368678; doi:10.3389/fpls.2026.1864757)

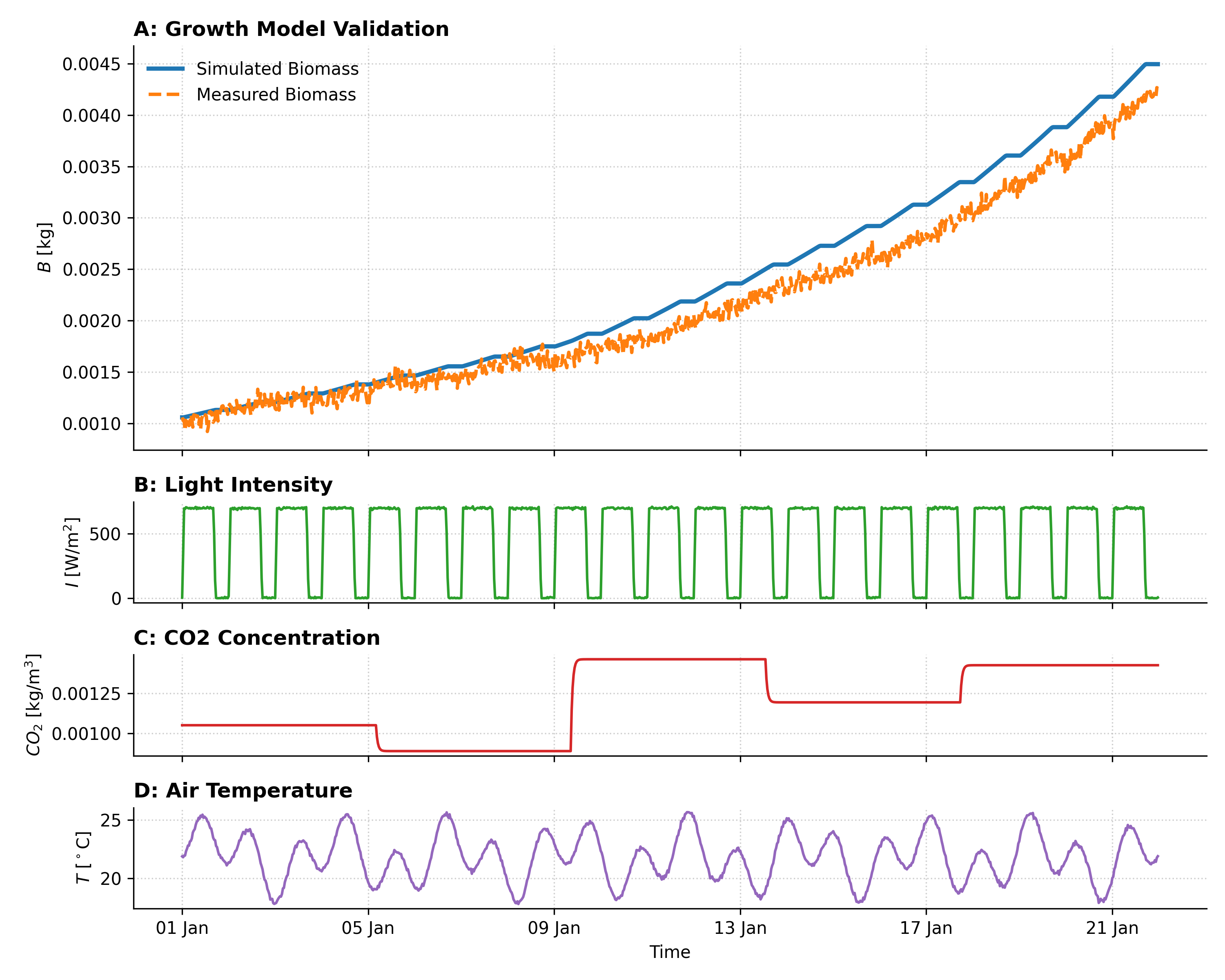

Supplement: Supplementary file 1 [file DataSheet1.zip › dt_code-main/estimation/parameter_estimation/results/pe_test_results.png]

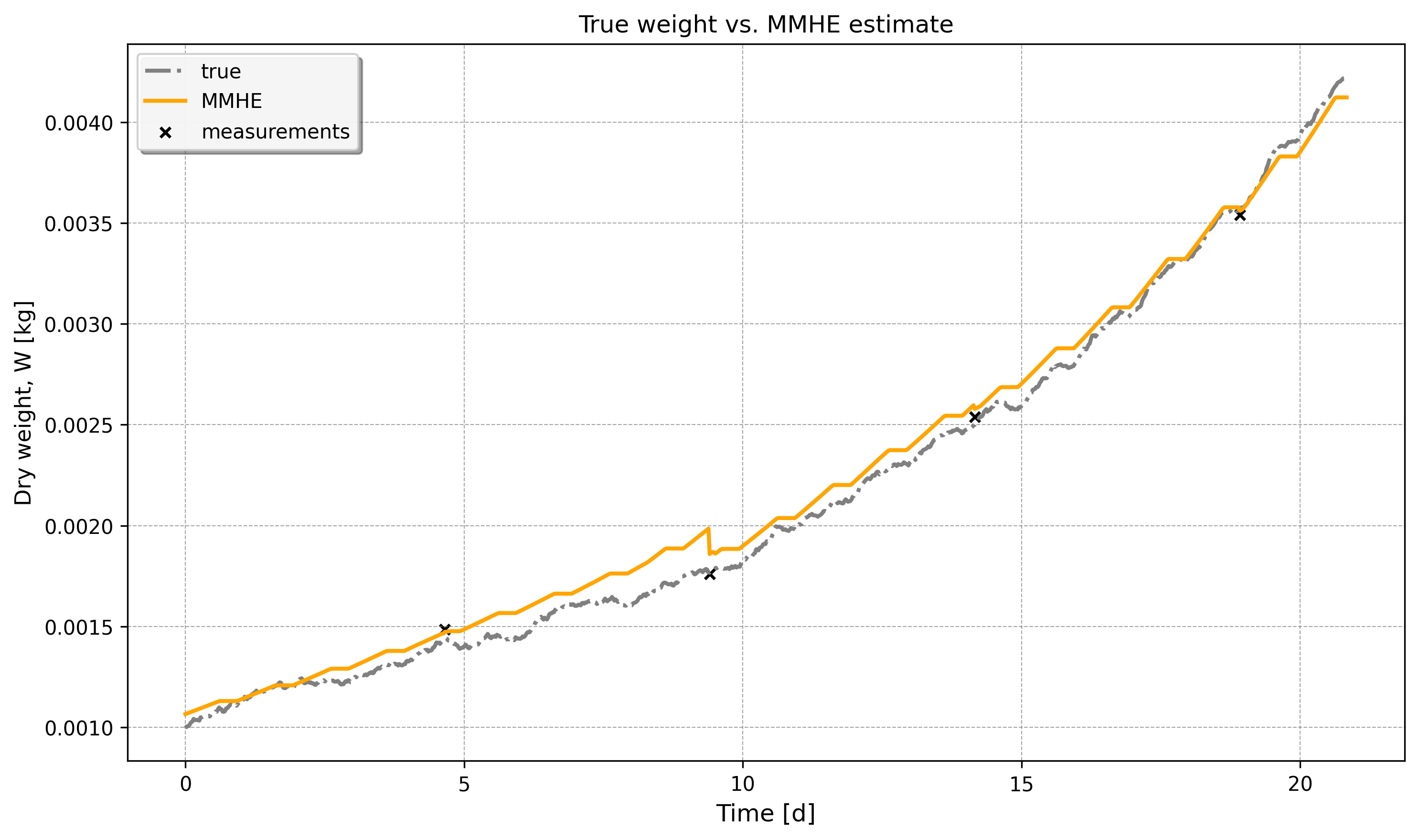

Supplement: Supplementary file 1 [file DataSheet1.zip › dt_code-main/estimation/state_estimation/results/mmhe_lettuce_results.png]
